# Insight into Preventing Global Dengue Spread: Nanoengineered Niclosamide for Viral Infections

**DOI:** 10.1021/acs.nanolett.4c02845

**Published:** 2024-08-28

**Authors:** N. Sanoj Rejinold, Geun-woo Jin, Jin-Ho Choy

**Affiliations:** † Intelligent Nanohybrid Materials Laboratory (INML), College of Medicine, Dankook University, Cheonan 31116, Republic of Korea; ‡ Institute of Tissue Regeneration Engineering (ITREN), Dankook University, Cheonan, 31116, Republic of Korea; § R&D Center, Hyundai Bioscience Co. LTD., Seoul 03759, Republic of Korea; ∥ Division of Natural Sciences, The National Academy of Sciences, Seoul 06579, Republic of Korea; ⊥ Tokyo Tech World Research Hub Initiative (WRHI), Institute of Innovative Research, Tokyo Institute of Technology, Yokohama 226-8503, Japan

**Keywords:** Dengue virus (DENV), Antiviral therapy, Nanoengineered niclosamide, Perspectives

## Abstract

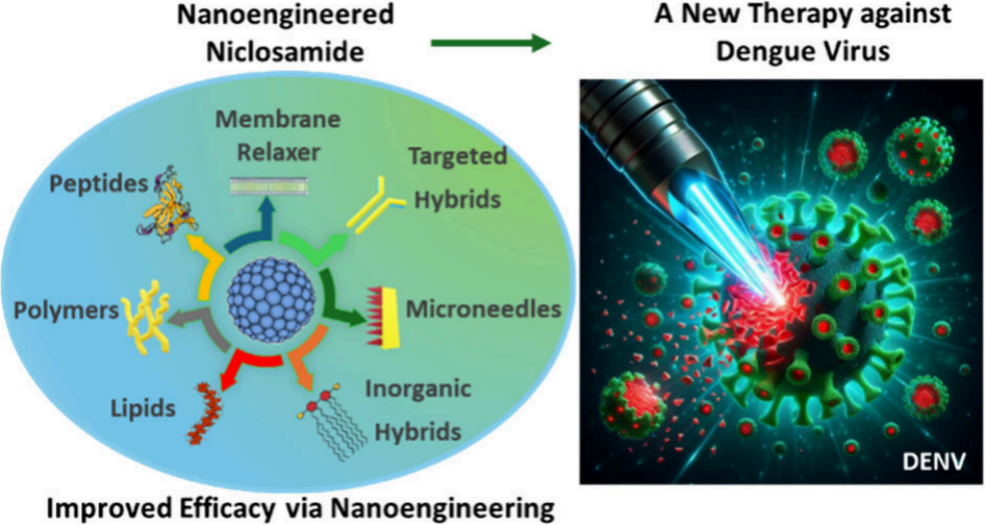

Millions of cases of dengue virus (DENV) infection yearly from *Aedes* mosquitoes stress the need for effective antivirals. No current drug effectively combats dengue efficiently. Transient immunity and severe risks highlight the need for broad-spectrum antivirals targeting all serotypes of DENV. Niclosamide, an antiparasitic, shows promising antiviral activity against the dengue virus, but enhancing its bioavailability is challenging. To overcome this issue and enable niclosamide to address the global dengue problem, nanoengineered niclosamides can be the solution. Not only does it address cost issues but also with its broad-spectrum antiviral effects nanoengineered niclosamide offers hope in addressing the current health crisis associated with DENV and will play a crucial role in combating other arboviruses as well.

Dengue virus (DENV) is one of the major mosquito-borne infectious diseases among Zika virus disease, Chikungunya, West Nile virus (WNV), Rift Valley fever (RVF), and Yellow fever (Table 1).^1^ Also DENV was found to be the most significant mosquito-borne disease globally in terms of incidence and geographical spread. This fastest-growing mosquito-borne disease is estimated to have ∼400 million infections annually according to the World Health Organization (WHO), making DENV a major public health concern.^2^ It accounts for a substantial proportion of mosquito-borne disease cases, particularly in urban and semiurban areas in tropical and subtropical regions. While malaria has historically been one of the most significant mosquito-borne diseases in terms of mortality,^3^ dengue’s incidence has increased dramatically in recent decades. The global effort to control malaria has seen some success, but dengue continues to spread rapidly, particularly in densely populated regions.^4^ DENV accounts for a significant and growing proportion of mosquito-borne infectious diseases, especially in tropical and subtropical regions where *Aedes* mosquitoes thrive.^5^ Its high incidence and potential for severe disease make it a critical focus for public health efforts.

**Table 1:** Comparing Diseases That Have Characteristics and Symptoms Similar to Those of Dengue Fever

disease	pathogen	vector	common symptoms	geographical distribution	ref
Chikungunya	Chikungunya virus	*Aedes aegypti*, *Aedes albopictus*	high fever, joint pain, headache, rash	Africa, Asia, Americas	(10)
Zika virus disease	Zika virus	*Aedes aegypti*, *Aedes albopictus*	fever, rash, joint pain, conjunctivitis	Americas, Africa, Southeast Asia	(11)
Yellow fever	yellow fever virus	*Aedes aegypti*	fever, chills, headache, muscle pain, jaundice	Africa, South America	(12)
Rift Valley fever	Rift Valley fever virus	*Aedes* species	fever, muscle pain, headache, conjunctivitis	Africa, Middle East	(13)
West Nile virus	West Nile virus	*Culex* species	fever, headache, body aches, rash	Africa, Europe, North America	(14)
Malaria	*Plasmodium* spp.	*Anopheles* species	fever, chills, headache, nausea, vomiting	Sub-Saharan Africa, Southeast Asia	(15)

Each year, approximately 400 million infections are caused by the dengue virus (DENV), which is transmitted via *Aedes* mosquitoes. Symptoms typically manifest 3–14 days postinfection and include headache, vomiting, fever, rash, and myalgia. Severe cases can result in central nervous system impairment, organ failure, plasma leakage, and even fatal dengue hemorrhagic fever and dengue shock syndrome.^6^ Over the past two decades, there has been an approximately 10-fold rise in the annual incidence of DENV infections worldwide, soaring from 500000 to 5.2 million cases (Figure 1). The relentless spread of the disease since early 2023, coupled with the surge in unexpected cases of DENV infections, has resulted in over 5 million reported cases and more than 5000 dengue-related fatalities across 80 or more countries. Of these cases, over 4.1 million DENV infections were concentrated in South America, with Brazil alone contributing to over 3 million cases.^7^ In Brazil, the situation was exacerbated by the continued spread of the infection since early 2023 and an unprecedented explosive increase in cases in 2024. On February 8, 2024, Rio de Janeiro, Brazil, declared a public health emergency due to a dengue fever epidemic, just days before the Carnival celebrations were scheduled to begin across the country. On February 27, 2024, Peru declared a health emergency in response to the rapidly increasing number of cases of dengue fever throughout the South American nation. The Health Minister of Peru stated that over 31000 dengue cases were reported in the first 8 weeks of 2024, resulting in 32 deaths. On March 27, 2024, Puerto Rico’s Department of Health declared a public health emergency in response to a surge in DENV cases in the territory. Puerto Rico has already recorded at least 549 cases of dengue and 340 dengue-related hospitalizations in 2024, compared to a total of 1293 cases in 2023. Despite the alarming escalation of DENV outbreaks,^8^ the development of effective antiviral treatments remains elusive.

**Figure 1:**
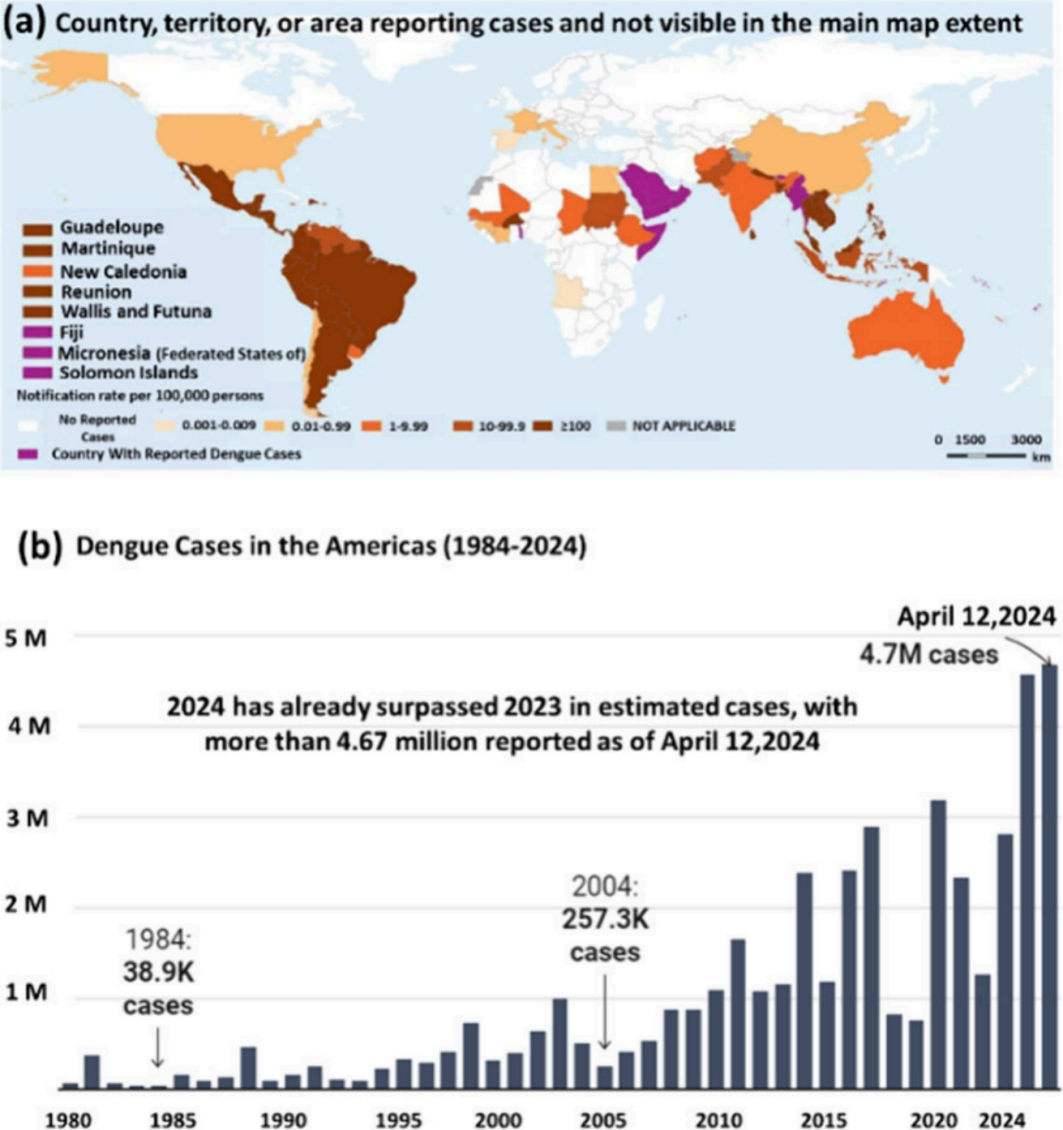
(a) Countries or regions where indigenous dengue cases are reported from November 2022 to November 2023 (Data source: World Health Organization (21 December 2023); Disease Outbreak News; Dengue–Global situation Available at: https://www.who.int/emergencies/disease-outbreak-news/item/2023-DON498). (b) Increase in dengue cases in the Americas over time, as reported by the Pan American Health Organization (PAHO), 2024.

This review was written to propose the use of nanoengineered niclosamide as a method to combat the global spread of the dengue virus. Niclosamide, a drug previously utilized for its antiparasitic properties,^9^ shows promise as an effective antiviral agent against DENV. However, its low bioavailability has prevented its clinical application as an antiviral drug. In this context, we discuss the urgent necessity for nanoengineering strategies to address the escalating dengue crisis. By conducting a comprehensive analysis of the current landscape of DENV infections and the challenges encountered in antiviral drug development, this review emphasizes the importance of exploring nanobased approaches such as nanohybrid technology to expedite the development of effective antiviral interventions against DENV infections.

## Characteristics of DENV

DENV is classified into four serotypes: DENV-1, DENV-2, DENV-3, and DENV-4. Although they exhibit genetic differences, with approximately 65% of their genome being shared, infection with any of these serotypes leads to the manifestation of similar clinical symptoms and the same disease.^16^ Following infection by a specific serotype, an individual acquires immunity against that particular serotype, inducing type-specific antibodies and leading to homotypic immunity against the infecting serotype. However, the immunity acquired from one serotype does not fully protect against infections caused by other serotypes.^17^ The immunity across different serotypes lasts for around 6 months and increases the possibility of severe dengue during subsequent infections, primarily due to antibody-dependent enhancement (ADE).^18^ ADE occurs when non-neutralizing antibodies that react to multiple serotypes of the virus facilitate viral entry into target cells by binding to Fc (fragment crystallizable) receptors, ultimately causing severe dengue shock syndrome.^19^ Consequently, to effectively combat DENV infection, interventions such as therapeutics and vaccines must target all four serotypes, as this is a critical requirement for both treatment and prevention strategies. However, the characteristics of DENV pose challenges in the development of vaccines and therapeutics, contributing to the absence of fully approved dengue vaccines and treatments.

## DENV Antivirals under Development

The dengue antivirals currently under development aim to directly inhibit the viral protein activity. Given that blocking the NS3–NS4B interaction inhibits DENV replication, clinical trials are underway for antivirals utilizing this mechanism. JNJ-1802 impedes viral replication by preventing complex formation between NS3 and NS4B, thereby inhibiting the formation of new viral RNA. Moreover, NITD-688 directly binds to NS4B.^20^ However, the absence of the NS4B crystal structure, likely due to its dynamic nature, poses challenges. Additionally, the low sequence homology of dengue NS4B with other viruses, such as Zika virus (ZIKV), West Nile virus (WNV), hepatitis C virus (HCV), Yellow Fever Virus (YFV), and Japan encephalitis virus (JEV), hinders the development of broad-spectrum inhibitors targeting NS4B. The NS4B mutations may lead to resistance against antivirals, as Goethals et al. identified resistance mutations within NS4B for JNJ-1802, with the emergence of the first persistent mutation (V91A) after 20 passages.^21^ The development of AT-752 targeting the RdRp function of NS5 showed potent antiviral activity against DENV-2, DENV-3, ZIKV, WNV, and YFV *in vitro*. Despite preclinical efficacy in reducing viremia and improving survival in DENV2-infected hamsters, phase 1 and phase 2 trials assessing safety and antiviral activity were discontinued due to a prioritization adjustment in the development pipeline (Table 2).

**Table 2:** Status of Dengue Antiviral Development

subtance name	developer	current phase of development	additional information
JNJ64281802/JNJ-1802	J&J	phase II clinical trials	multinational preventive clinical trials in phase II are currently in progress
			scope/details: planned enrollment of 1850 participants across multiple nations, including Brazil, Malaysia, the Philippines, Thailand, Colombia, and Peru
			study timeline: initiated enrollment on February 22, 2023, with an anticipated completion date of May 22, 2025; participant recruitment is ongoing
			primary end point: incidence of dengue virus infection
NITD-688 (EYU688)	Novartis	phase II clinical trials	conducting phase II trials in Singapore
			scope/location: Anticipated enrolment of 108 participants, exclusively in Singapore
			study timeline: expected to commence on January 12, 2024, concluding on January 29, 2025, with ongoing participant recruitment
			critical inclusion/exclusion criteria: enrollment is required within 48 h of high fever onset, confirmed dengue infection, and exclusion of patients with severe dengue
			primary end point: viral load assessment from baseline to 48 h post-treatment.
AT-752	Atea	phase II clinical trials (suspended)	suspension of trials attributed to strategic reprioritization due to budgetary constraints and development challenges

## Niclosamide-Based Antiviral Drugs to Overcome the Limitations of Currently Developing Dengue Antiviral Drugs

As discussed in the previous section, currently there is no approved dengue antiviral available. Additionally, the investigational antivirals, known as direct-acting antivirals (DAAs), target the mechanisms directly involved in DENV infection.^22^ However, DAAs cannot provide comprehensive responses to arbovirus infections transmitted by mosquito bites. For instance, mosquitoes transmitting DENV also transmit Chikungunya virus; however, while DAAs are effective against viruses belonging to the Flaviviridae family having structural similarities with DENV, they may not be effective against viruses belonging to other families, such as Togaviridae, to which the Chikungunya virus belongs.^23^ Overcoming the public health crisis caused by DENV ultimately requires a broader antiviral approach encompassing others, thus highlighting the importance of a comprehensive antiviral effect against arboviruses.^24^ Furthermore, DAAs may lead to the development of resistance to viral mutations, as demonstrated in the study of Goethals et al.^21^ Considering that viruses hijack cellular pathways to create a favorable environment for replication, host-directed antivirals (HDAs) can address these challenges by targeting host cells, rather than the virus itself. Arboviruses, including DENV, often exploit host factors that are similar to their replication. Therefore, HDAs have a significant potential to effectively combat infections caused by arboviruses. Furthermore, HDAs target host factors of the virus that are less prone to mutations, reducing the risk of resistance development.^25^

Niclosamide has demonstrated antiviral efficacy not only against DENV but also against various viruses within the Flaviviridae family, including ZIKV, WNV, YFV, JEV, and HCV, *in vitro*.^26^ It is known to exert broad-spectrum antiviral activity by interfering with the cellular machinery utilized by flaviviruses during infection.^26^ Flavivirus particle maturation, which is crucial for viral infectivity, occurs in the low-pH environment within endosomes. Essentially, flaviviruses, including DENV, exploit the endosomal system of cells for maturation.^27^

Within infected cells, immature icosahedral virions emerge from the endoplasmic reticulum through a budding process initiated by the lateral interaction of prM and E glycoprotein heterodimers. As virions undergo exocytosis via the trans-Golgi network, the acidic conditions trigger significant particle reorganization.^28^ The latter involves the formation of head-to-tail dimers by the E protein and the cleavage of prM into globular pr and transmembrane M proteins by furin.^29^

Kao et al. demonstrated the antiviral efficacy of niclosamide against DENV-2.^30^ Using *in vitro* cell models of DENV infection, they confirmed that niclosamide efficiently suppressed the expression of viral proteins and markedly delayed the viral release. Moreover, niclosamide neutralized the acidic pH environment within endosomes during DENV infection by inhibiting dsRNA replication and viral release. Similarly, Jung et al. demonstrated the antiviral efficacy of niclosamide against DENV-1, -2, -3, and -4.^31^

**Figure 2:**
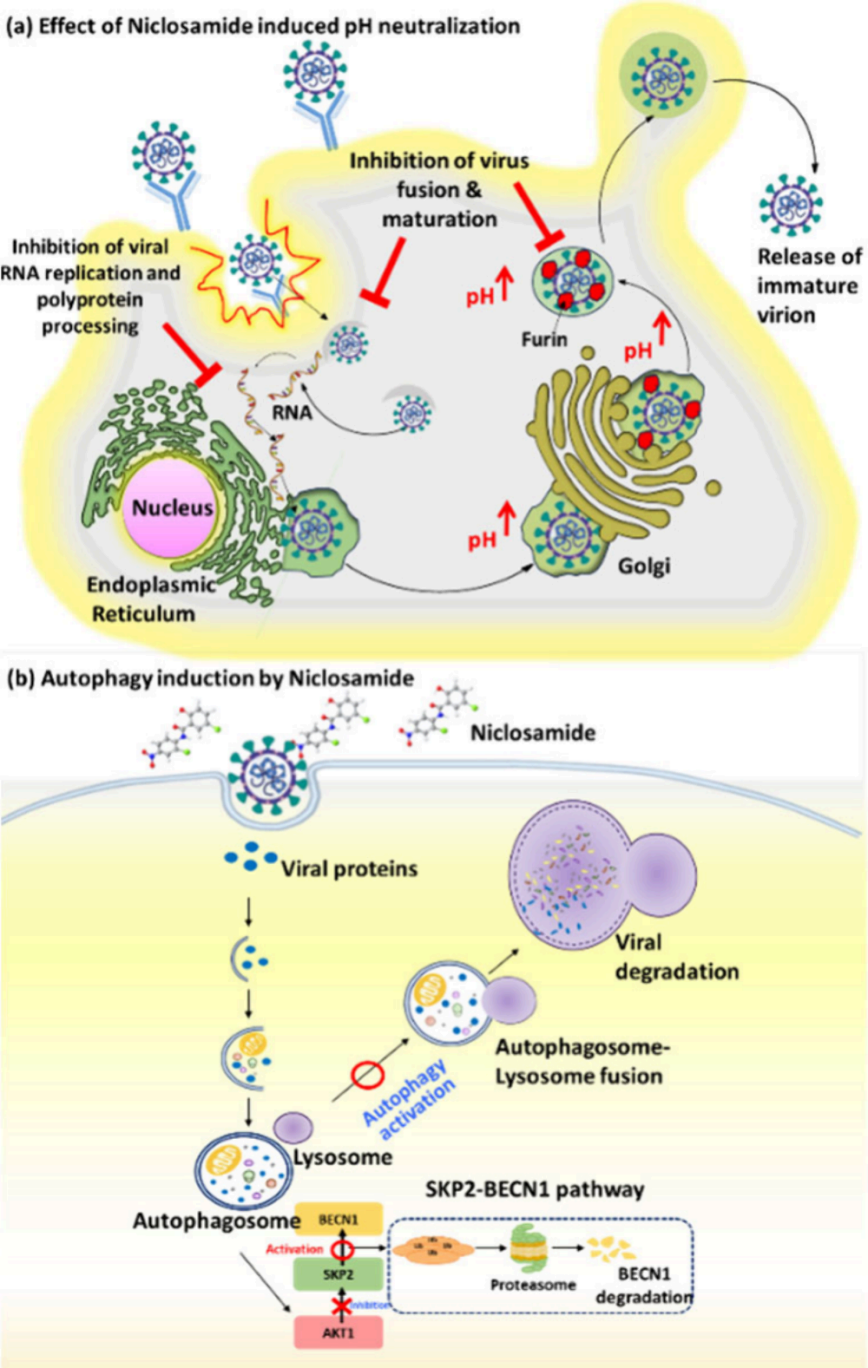
(a) A diagram illustrating the life cycle of flaviviruses and the proposed antiviral mechanism of niclosamide. Niclosamide inhibits various stages of the DENV life cycle by neutralizing acidic intracellular organelles, thereby inhibiting viral RNA replication, polyprotein processing, virus fusion, and the maturation of new virions. Adapted from ref (31). Available under CC-BY [4]. Copyright 2019 Nature. (b) SKP2 inhibitor niclosamide reduces virus replication through autophagy. Adapted from ref (47). Available under a CC-BY [4]. Copyright 2019 Nature.

Niclosamide-induced neutralization interfered with the pH-dependent DENV maturation process, leading to the liberation of immature and noninfectious virus particles (Figure 2a). Although these findings elucidate the antiviral mechanism of niclosamide against DENV, they do not fully explain its antiviral effects against various viral families beyond Flaviviridae. The extensive antiviral effects of niclosamide beyond the Flaviviridae family may also involve autophagy.^32^ Autophagy plays a role in the innate immune response against virus-infected cells, indicating a potential mechanism through which niclosamide could exert its antiviral effects against viruses beyond Flaviviridae. In 2019 and 2021, Gassen et al. demonstrated that niclosamide demonstrates antiviral efficacy against both Middle East respiratory syndrome coronavirus (MERS-CoV) and severe acute respiratory syndrome coronavirus 2 (SARS-CoV-2) through SKP2 inhibition, inducing autophagy (Figure 2b).^33^ Chen and Smartt demonstrated the antiviral effects of autophagy inducers against DENV.^34^ They explored the involvement of the autophagy pathway in the *Aedes aegypti* cell line Aag-2 by employing small molecules, such as rapamycin and 3-methyladenine (3-MA), to modulate autophagy. Rapamycin, known for its ability to inhibit the mammalian target of rapamycin complex 1 (mTORC1), triggers autophagy.^35^ Conversely, 3-methyladenine (3-MA), a targeted inhibitor of phosphoinositide 3-kinase (PI3K), disrupts the formation of autophagosomes.^36^ Notably, treatment with 3-MA did not significantly affect the DENV titer. These findings suggest that cell modulation to induce subsequent autophagy could serve as a potential treatment strategy for DENV infections.

## The Hurdles to Repurposing Niclosamide as an Antiviral and the Nanoengineering Techniques to Overcome Them

Despite its broad-spectrum antiviral activity against several viruses, including DENV, niclosamide has not been utilized as an antiviral drug. One of the reasons is that its physical and chemical properties have acted as obstacles to drug repurposing. Niclosamide is a yellowish crystalline solid with limited solubility in water (typically approximately 5–8 mg/L at 20 °C). It is sparingly soluble in ether and dissolves in solvents such as ethanol (22 mM) and DMSO (10 mM). Its poor solubility in water is a major factor contributing to its poor bioavailability (approximately 10%). Furthermore, previous research has shown that niclosamide is known to have intermolecular π–π interactions, which further exacerbate its poor bioavailability, thus restricting its medical applications.

In addition to this, the other critical disadvantage that makes niclosamide difficult to repurpose as an antiviral drug is that it is rapidly metabolized in the intestines and liver. Fan et al. revealed that niclosamide is rapidly metabolized through glucuronidation in the liver and intestines. Therefore, their strategy to increase the bioavailability of niclosamide is to inhibit niclosamide glucuronidation in both the liver and intestines.^37^ However, enhancing drug bioavailability by coadministering enzyme inhibitors can lead to a wide range of drug–drug interactions, which may limit the range of patients who can safely be prescribed the drug and raise safety concerns.^38^

By using nanoengineering to effectively deliver niclosamide to the digestive system, the loss of niclosamide due to metabolism can be minimized. In fact, studies that have used nanoengineering to deliver niclosamide orally have shown the potential of this approach (Table 3).

**Table 3:** Nanoscale Technologies in the Formulation of Niclosamide (Long-Lasting Solubility and Bioavailability Issues)

nanoscale technologies	descriptions	characteristics	viral family/remarks	improved performance metrics	integration of nanoscience/nanotechnology	ref
solid lipid nanoparticles (SLNs)	nanoparticles composed of solid lipids that encapsulate niclosamide	particle size 204.2 ± 2.2 nm, polydispersity index 0.328 ± 0.02 and zeta potential –33.16 ± 2 mV; entrapment efficiency and drug loading capacity were 84.4 ± 0.02% and 5.27 ± 0.03%, respectively.	this formulation could be beneficial for DENV infection	increased bioavailability and prolonged drug release; improved efficacy in preclinical studies	the chemical interactions likely involve hydrogen bonding interaction and van der Waals one between drug and lipid molecules, which stabilize the formulation and ensure uniform drug distribution within nanoparticles	(40)
liposomes	lipid bilayer vesicles encapsulating niclosamide, protecting it from degradation	the liposomal niclosamide is based on egg phosphatidylcholine (Egg PC), cholesterol; distearoylphosphatidylethanolamine (DSPE)-PEG1000 and DSPE-PEG750 were from Avanti Polar Lipids, with particle size ∼200 nm	Coronaviridae; such formulation could be used for DENV infections too	potency against SARS-CoV-2 infection in cells (Vero E6 and ACE2-expressing lung epithelium cells)	this encapsulation is stabilized by van der Waals forces, hydrogen bonding, and hydrophobic interactions, which mimic cell membranes and improve drug delivery; the increased surface area at the nanoscale facilitates efficient absorption and release within target cells, overcoming niclosamide’s solubility challenges and boosting its antiviral effectiveness	(41)
polymeric micelles	amphiphilic block copolymers forming micelles that solubilize niclosamide	the inhibitory effect of NIC on Wnt/β-catenin and Notch signaling pathways was potentiated by the NIC-NP formulation. The particle size was ∼437.2 ± 70.25 nm	DENV (the activation of the Wnt/β-catenin pathway by dengue virus can enhance viral replication; the pathway’s activation promotes a cellular environment conducive to viral replication, providing the necessary resources and conditions for efficient viral propagation)	improved bioavailability, enhanced therapeutic effect in animal models	at the nanoscale, the interaction between niclosamide molecules and pluronic copolymer involves hydrogen bonding and hydrophobic interactions, which encapsulate drug molecules within the nanoparticle matrix, allowing a sustained and controlled release of niclosamide over an extended period; consequently, it improves bioavailability by targeted delivery to specific tissues and cells, such as the liver in the case of hepatocellular carcinoma (HCC) treatment; such advancement in nanoscience gives rise to an improvement of therapeutic efficacy of niclosamide	(42)
protein hybrids	polyethylene glycol (PEG) coated bovine serum albumin (BSA) stabilized niclosamide (NIC) nanoparticles (NPs) (∼BSA-NIC-PEG NPs)	improved solubility for the niclosamide hybrid, particle size was ∼120 nm	such a repurposed niclosamide could be effective toward DENV infections	PK study showed significant improvement in *C*_max_ and *T*_max_ of niclosamide from hybrid in comparison to pure niclosamide alone	niclosamide interacts with BSA via ionic and hydrogen bonding interactions and further sterically stabilized by PEG, eventually enhancing the solubility and thereby increasing the PK parameters	(43)
nanocrystals	pure niclosamide reduced to nanometer-sized crystals, increasing surface area	the niclosamide nanocrystals (160 nm) were based on Tween-80 and 1,2-distearoyl-*sn*-glycero-3-phosphocholine (DSPC) enhancing dissolution rate, improved absorption	Coronaviridae	repurposed niclosamide nanocrystals can be used for DENV therapy	in the niclosamide dry powder formulation, Tween-80 and DSPC interact with niclosamide nanocrystals primarily through van der Waals forces and hydrophobic interactions, stabilizing the particles and preventing aggregation; this surface modification enhances the dispersibility and maintains the structural integrity of the microparticles during and after spray freeze-drying	(44)
inorganic hybrid nanoparticles	niclosamide is loaded into MgO and coated with hydroxy propyl methyl cellulose	∼200 nm sized inorganic hybrids improved pharmacokinetics and targeted delivery toward SARS-CoV-2	Coronaviridae	increased bioavailability, reduced required dosage, improved therapeutic outcomes	since niclosamide undergoes deprotonation in the presence of basic magnesium oxide (MgO) nanoparticles with a positive zeta potential, thus formed niclosamide-MgO nanohybrid can be stabilized via ionic-bonding interaction; upon hybridizing with mucoadhesive hydroxypropyl methylcellulose (HPMC) to form niclosamide-MgO-HPMC nanohybrid; the niclosamide’s solubility and intestinal permeability can be greatly enhanced; these interactions collectively facilitate the effective delivery and absorption of niclosamide without changing its metabolic pathway, thereby improving not only the solubility, but also the PK parameters and ultimately enhanced efficacy	(45)

One thing to note here is that countries or regions experiencing widespread viral outbreaks often lack adequate medical support, making the development of oral treatments more crucial than injectable antiviral medications. Moreover, there is a need for a way to ensure that medication can be safely administered to as many patients as possible without issues such as drug–drug interactions. Nanoengineering is a strategy that can meet this need.

Bhattacharyya et al. synthesized CP-NIC by conjugating niclosamide to a genetically encoded elastin-based chimeric polypeptide (CP). CP-NIC formed cylindrical nanoparticles through a self-assembly process, with an average length measured as 74 ± 10 nm (*n* = 10) and an average diameter (DTEM) of 12.5 ± 3.5 nm, as determined by cryo-TEM. Pharmacokinetic comparisons were made by injecting samples intravenously in mice, revealing that while niclosamide had a terminal half-life and AUC of 1.0 ± 0.22 h and 3.3 ± 1.3 μg/(mL h), respectively, CP-NIC exhibited a terminal half-life of 4.2 ± 1.34 h and a plasma AUC of 36.9 ± 7.34 μg/(mL h).^39^

Lin et al. manufactured nanosized niclosamide with enhanced bioavailability through a colloidal dispersion method using electrospray.^46^ While the original niclosamide suspension was turbid, the nanosized niclosamide (nano-NI colloidal dispersion) exhibited clarity with a yellowish color. Scanning electron microscopy (SEM) images revealed that niclosamide in this nanosuspension had an average particle diameter and length of 105–21 and 493–151 nm, respectively. Upon oral administration of this nanosuspension to rats, the maximum plasma concentration of niclosamide was observed at 5 min, and bioavailability was confirmed to be 25%.^46^

Jara et al. aimed to enhance the bioavailability of niclosamide by preparing `amorphous niclosamide. They mixed niclosamide, PVP-VA, and TPGS in a 60:35:5 ratio and extruded the mixture (at a feed rate of 3 g/min at a screw speed of 50 rpm). Subsequently, granules were obtained by milling the extrudates with a Fitz mill. This process not only increased the apparent solubility of niclosamide from 6.6 ± 0.4 to 481.7 ± 22.2 μg/mL in fasted-state simulated intestinal fluid (FaSSIF) but also improved its oral bioavailability by 2.6-fold in Sprague–Dawley rats when administered orally as a suspension.^48^

Gan et al. used the solvent evaporation method to prepare niclosamide nanoparticles. In this process, the niclosamide and PCEC ((poly(ε-caprolactone, ε-CL)-poly(ethylene glycol)-poly(ε-CL)) solutions were dripped into an aqueous solution containing SDS (Sodium dodecyl sulfate), and niclosamide nanoparticles were formed by using a rotary evaporator. This method yielded uniform nanoparticles with an average size of approximately 172 ± 2 nm (polydispersity index [PDI] = 0.120 ± 0.06). The niclosamide nanoparticles exhibited slow cumulative release behavior compared to free niclosamide, which showed rapid release. Additionally, these nanoparticles demonstrated improved water solubility and dispersion in aqueous solutions. Therefore, niclosamide nanoparticles can provide a uniform injectable dosage with an aqueous form suitable for *in vivo* delivery.^49^

A recent study by Choy et al. focused on the antiviral effectiveness of niclosamide against SARS-CoV-2, with the aim of repurposing it as an antiviral agent. They successfully enhanced the bioavailability of niclosamide (NIC) by formulating it using inorganic–organic compounds (NIC-MgO-HPMC) via nanohybrid technology. The inorganic compound was found to disrupt the π–π interactions of niclosamide through hydrogen bonding with niclosamide molecules. Dissolution experiments revealed an improved cumulative release of niclosamide (55% for niclosamide and 97% for NIC-MgO-HPMC), leading to an enhanced bioavailability. This formulation demonstrated a statistically significant reduction in SARS-CoV-2 viral load and improved pneumonia lesions compared with niclosamide alone. Consequently, this research advanced to clinical trials, where both the safety and efficacy against SARS-CoV-2 were demonstrated in humans.^50^

**Figure 3:**
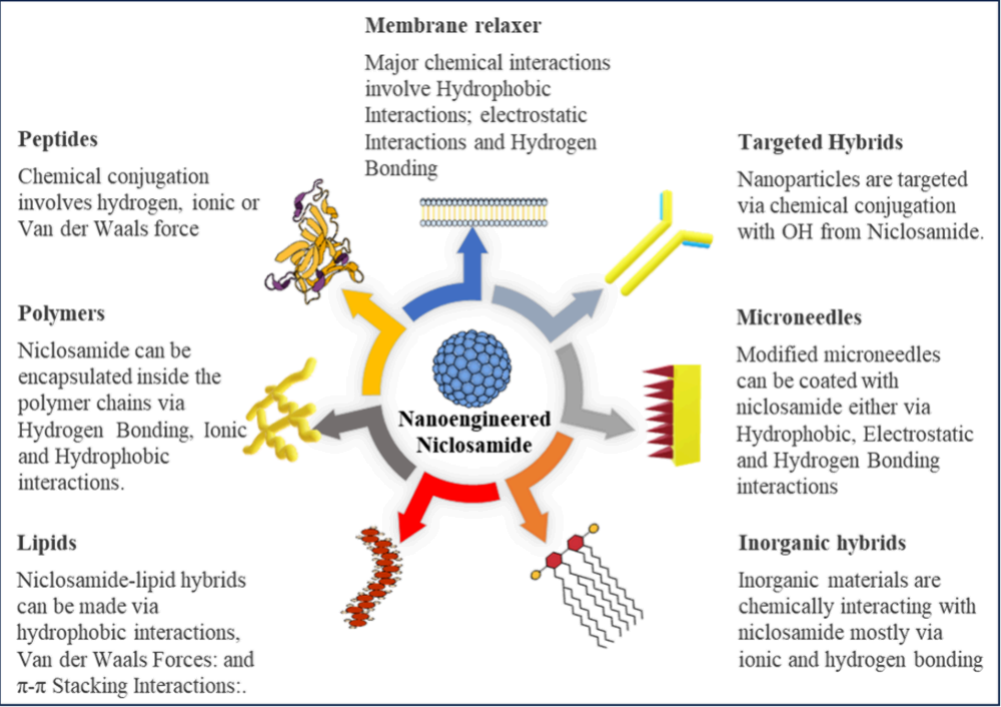
Various chemical approaches in nanoengineered niclosamides against DENV viral infections.

Similarly, a lipid nanoparticle formulation of niclosamide (nano-NCM) was effective in inhibiting SARS-CoV-2 replication *in vitro*.^41^ Further, a lithocholic acid-tryptophan conjugate (UniPR126)-based mixed micelle as a nanocarrier was demonstrated to specifically deliver niclosamide to a prostate cancer site via the EphA2 receptor.^51^ These recent studies clearly shed light on the fact that rationally designed nanoengineered niclosamide could be beneficial for DENV therapy (Figure 3). Additionally, the rational development of such efforts must maintain the unique properties that are essential for nanomaterials for viral diseases (Table 4).

**Table 4:** Unique Properties Required for Nanomaterials for Applications in Virus-Related Diseases

viral diseases	nanomaterials	properties	descriptions	applications	ref
COVID-19, H1N1, HIV	gold nanoparticles	high surface area, and optimum size in the range <50 nm	large surface area to volume ratio for increased interaction with viral particles	enhances binding and detection sensitivity in diagnostic assays	(52)
HIV, HBV, HSV, DENV, etc	chitosan-based nanoparticles	biocompatibility with an optimum size in the range of 50 to 700 nm with controllable size	nontoxic and compatible with biological systems, precisely controlled size and shape for optimal interaction with viruses	safe for use *in vivo* for drug delivery and therapeutic applications against various infectious diseases as listed, improved efficacy in enhanced drug delivery, targeted delivery, sustained release, immunomodulatory effects, mucosal adhesion, etc.	(53)
COVID-19	PEG-PCL-loaded with remdesivir	functionalization	ability to attach functional groups or biomolecules	targeted delivery and improved specificity for antiviral drug delivery and diagnostics	(54)
COVID-19, DENV, HIV diseases	QDs with ultrasmall size	optical properties	unique optical characteristics such as fluorescence or plasmon resonance	used in imaging, diagnostic assays, and biosensors	(55)
COVID-19, DENV, etc.	carbon nanomaterials such as fullerenes	electrical properties	high electrical conductivity for enhanced signal transduction	electrochemical sensors for virus detection and monitoring	(56)
avian influenza virus	iron oxide nanoparticles with size 30–300 nm	magnetic properties	magnetic responsiveness for external control and targeting	magnetic nanoparticles for targeted drug delivery and magnetic resonance imaging (MRI)	(57)
cowpea chlorotic mottle viruses	cationic lignin nanoparticles with size ∼122 nm	antiviral activity	intrinsic ability to inhibit viral replication or neutralize viruses	direct use as antiviral agents or coatings on surfaces to prevent viral spread	(58)
COVID-19, HIV, EBOV disease, etc	stimuli sensitive or targeted nanoparticles	controlled release	ability to release therapeutic agents in a controlled manner	sustained and targeted delivery of antiviral drugs	(59)
COVID-19	PDZ2-conjugated-PLGA nanoparticles with a size of 235 nm	stability	chemical and physical stability in biological environments	maintains functionality and efficacy in physiological conditions	(60)

Additionally, it is worth mentioning that there have been attempts to dengue virus treatment using various nanomaterials paving a way for exploring the nanotools for DENV infection (Table 5)

**Table 5:** Cases of Dengue Virus Treatment Using Nanomaterials

nanomaterials	treatment methods	pros	cons	effects	ref
gold nanoparticles (AuNPs)	conjugated with dengue antibodies for targeted delivery	high specificity and sensitivity, biocompatibility, easy to functionalize	high cost, potential toxicity at high doses	improved detection and targeted delivery of antiviral drugs, reducing viral load in infected cells	(63)
silver nanoparticles (AgNPs)	direct antiviral activity by disrupting viral envelope and inhibiting replication	broad-spectrum antiviral activity, biocompatible, easy synthesis	potential cytotoxicity to human cells, stability issues	reduced viral replication and enhanced antiviral activity *in vitro*	(64)
lipid nanoparticles	encapsulation of antiviral drugs for sustained release	enhanced drug stability and bioavailability, reduced side effects	complex manufacturing process, potential for immunogenicity	improved delivery and sustained release of antiviral drugs, leading to prolonged therapeutic effects	(65)
polymeric nanoparticles	encapsulation and controlled release of antiviral agents	high drug loading capacity, controlled release, reduced systemic toxicity	potential biodegradation issues, complex synthesis process	sustained release and improved therapeutic index of antiviral agents	(66)
silica nanoparticles	combined treatment of hydrophobic nanosilica with temephos in larvicidal test	high surface area, easily functionalized, biocompatible	potential toxicity, stability issues	independent toxic action without any additive effect	(67)
magnetic nanoparticles	colorimetric test for the detection of the NS1 protein of dengue virus, assisted by an immunoconjugate of magnetite (Fe_3_O_4_) nanoparticles coupled to anti-NS1 antibodies	targeted delivery, noninvasive guidance using external magnetic fields	potential cytotoxicity, complex synthesis and functionalization	simple, quick, and inexpensive, in situ biomolecular diagnostic test	(68)
quantum dots	used in diagnostics for rapid and sensitive detection of dengue virus	high sensitivity and specificity, multiplexing capability, strong fluorescent properties	potential cytotoxicity, expensive synthesis	improved detection sensitivity and specificity, allowing for rapid and accurate diagnosis	(69)
carbon nanotubes (CNTs)	functionalized with antiviral drugs for enhanced delivery	high surface area, strong mechanical properties, potential for targeted delivery	potential toxicity, complex functionalization process	enhanced delivery and binding of antiviral drugs, reducing viral replication and improving treatment outcomes	(70)

## Advanced Nanoengineered Niclosamide Applied in Humans through Clinical Trials

Although niclosamide has shown promise in laboratory studies as a potential antiviral agent against various viruses, including DENV, there are limited advanced nanoengineering strategies for niclosamide that have progressed to clinical trials. However, there are ongoing efforts to explore the efficacy of nanotechnology-applied niclosamide in humans through clinical trials.^50^

For example, Parikh et al. conducted a phase 1 clinical trial involving patients with castration-resistant prostate cancer, in which they administered a novel formulation of niclosamide (PDMX1001 capsules; detailed information regarding PDMX1001 was not available in the published literature). The primary purpose of this study was to assess the toxicity profile and plasma concentrations of niclosamide following the administration of PDMX1001. Patients received escalating doses of PDMX1001, and both the peak and the trough levels of niclosamide were measured. Plasma samples were collected from three patients before (trough) and 1 h after (peak) the ingested 1200 mg of PDMX1001. The observed trough concentrations ranged from 0.31 to 0.65 μM (100.1–212.1 ng/mL), while peak concentrations ranged from 0.21 to 0.72 μM (70.0–236.4 ng/mL). Notably, there was no significant difference between the trough and peak levels of niclosamide, indicating that the drug achieved steady-state concentrations. The combination of PDMX1001 with abiraterone and prednisone was well tolerated by the patients, with diarrhea being the most frequently reported adverse effect. Remarkably, among the eight evaluable patients, five demonstrated a prostate-specific antigen (PSA) response, with two individuals achieving undetectable PSA levels along with a radiographic response.^61^

Choy et al. conducted a clinical trial targeting patients with COVID-19 to assess the enhanced bioavailability of niclosamide using a novel formulation named NIC-MgO-HPMC and decided to rename its commercial product to XAFTY upon approval. Out of the intended recruitment goal of 300 participants, plasma concentrations of niclosamide were measured in 20 patients who received 300 mg of NIC-MgO-HPMC and 18 patients who received 450 mg of NIC-MgO-HPMC. The study revealed dose-dependent profiles, with niclosamide plasma concentrations of 241.5 ng/mL for the 300 mg dose and 406.5 ng/mL for the 450 mg dose of NIC-MgO-HPMC at a time point of 3 h. Overall, NIC-MgO-HPMC was well tolerated.^50^ These research findings signify the realization of the medical application of nanoengineered niclosamide and demonstrate readiness for its implementation in dengue clinical trials.

## Future Perspectives

As we have seen earlier, niclosamide is an optimal drug for repurposing as a DENV antiviral, and the hurdle of low bioavailability can be overcome with nanoengineering techniques. Many of the nanoengineered niclosamide studies reviewed earlier demonstrate that repurposing niclosamide as an antiviral is feasible. However, to suppress the global spread of DENV, especially in Least Developed Countries (LDCs), it is essential to employ nanoengineering techniques that meet certain criteria. In other words, it is crucial to critically assess which nanoengineering technologies are practically applicable and beneficial for people during outbreaks of infectious diseases such as DENV infection.

First, it is advisable to exclude new chemical entities (NCEs) that involve the chemical conjugation of niclosamide. NCEs must go through the entire drug development process, which can take 12–15 years from discovery to market.^62^

Given this lengthy development time, using niclosamide as a new chemical entity with chemical conjugation would take too long. Therefore, to effectively address the current dengue threat, it is more suitable to use nanoengineering techniques that utilize niclosamide without chemical conjugation.

Second, it should be an orally administrable form of nanoengineered niclosamide. According to the WHO, half the world lacks access to essential health services.^71^ To effectively address the global dengue threat, injectable forms of niclosamide that require the assistance of healthcare providers in hospitals are not ideal. Instead, it should be an oral form of nanoengineered niclosamide that patients can take by themselves.

Third, it is advasible to prioritize nanoengineered niclosamide that has undergone human clinical trials. The translation from the lab to bench side involves numerous hurdles, including the use of excipients with proven safety, the feasibility of production in Good Manufacturing Practice (GMP) facilities, and stability. Nanoengineered niclosamide that has entered clinical trials has overcome such hurdles, and those proven to be safe after clinical trials can accelerate the application of nanoengineered niclosamide for DENV infection. Fortunately, as previously discussed, there are nanoengineered niclosamide formulations that have already undergone clinical trials and have been proven safe in humans.

Nanoengineered niclosamide meeting these criteria offers unparalleled advantages compared to other antiviral drugs, namely, its broad-spectrum antiviral activity extending beyond viral families. The development of broad-spectrum antivirals is crucial for comprehensively addressing public health threats. Dengue fever, among other infections caused by arboviruses transmitted by arthropod vectors, poses a significant epidemiological risk, particularly with a recent increase in the number of cases, complications, and severity. As indicated by Beltrán-Silva et al., the cocirculation of dengue, Chikungunya, and Zika poses a significant public health concern, owing to their transmission by the same vector. Additionally, there has been an increase in the number of cases of microcephaly related to the ZIKV, post-Chikungunya chronic joint disease, and severe dengue.^72^ As per the latest report, it was observed that there are 6953 Zika, 5225804 dengue, and 186,362 Chikungunya cases worldwide.

In regions where DENV infections are frequent, public health crises stem from the difficulty in distinguishing among the clinical characteristics of the most common arboviral infections, such as dengue, Zika, and Chikungunya. These diseases share similarities and are transmitted by the same type of mosquito, leading to simultaneous infections. Therefore, addressing DENV infections alone cannot resolve the public health crisis resulting from the proliferation of mosquitoes due to climate change. Consequently, there is an urgent need for broad-spectrum antiviral agents that are effective against Zika and Chikungunya viral infections, as well.

The effectiveness of niclosamide against not only flaviviruses, including DENV and ZIKV, but also togaviruses, such as the Chikungunya virus, positions it as a strong candidate for arbovirus response. High viral loads of DENV are closely associated with severe dengue, emphasizing the importance of antiviral treatment promptly at symptom onset. With the availability of broad-spectrum antivirals, such as niclosamide, which has improved bioavailability through nanoengineering, treatments can be initiated even before diagnosis, potentially mitigating disease severity.

Given these factors, nanoengineered niclosamide is considered highly necessary to effectively overcome public health crises. Nanoengineered niclosamide, with its broad-spectrum antiviral activity, is expected to be developed in a form different from that of existing antivirals. Since nanoengineered niclosamide holds promise for treating not only DENV but also other arboviruses, it appears necessary to apply new forms of clinical trials, such as “basket trials’, to validate its effectiveness against arboviruses. Although such trial formats have primarily been applied in cancer research, they are crucial for developing broad-spectrum antiviral approaches against arboviruses, particularly in the case of repurposing nanoengineered niclosamide. These new trial formats are expected to become a desirable model for responding to global health crises caused not only by DENV but also by arboviruses in the near future.
